# Long-term particulate matter exposure and risk of androgenetic alopecia: A nationwide cohort study

**DOI:** 10.1016/j.jdin.2026.06.008

**Published:** 2026-06-21

**Authors:** Jee Yoon Park, Jihee Nam, Jin Lee, Suna Kang, Jihye Heo, Hwamin Woo, Sung Won Kang, Chang-Hun Huh, Danbee Kang, Jung-Won Shin, Juhee Cho

**Affiliations:** a Department of Dermatology, Seoul National University Bundang Hospital, Seongnam, Republic of Korea; b Department of Clinical Research Design and Evaluation, SAIHST, Sungkyunkwan University, Seoul, Republic of Korea; c Center for Clinical Epidemiology, Samsung Medical Center, Seoul, Republic of Korea; d Korea Environment Institute, Sejong, Republic of Korea

**Keywords:** androgenetic alopecia, environmental risk factors, hair follicle inflammation, oxidative stress, particulate matter (PM_2_._5_, PM_10_)

**Figure:**
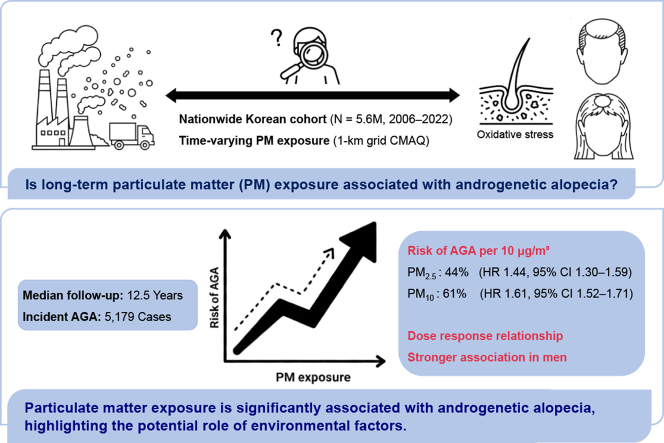

*To the Editor:* Androgenetic alopecia (AGA) is the most common form of hair loss, characterized by progressive miniaturization of hair follicles in androgen-sensitive areas. Although its pathogenesis is multifactorial, the role of environmental factors remains underexplored. Particulate matter (PM), a major component of air pollution, activates aryl hydrocarbon receptor (AhR) and NF-κB signaling, inducing oxidative stress and inflammation in the skin and hair follicle.^1^ However, large-scale epidemiological evidence on the association between chronic PM exposure and AGA risk is lacking.

We conducted a nationwide cohort study using the Korean National Health Insurance Service database (2002-2006). We included 5,591,500 adults (mean age, 44.4 years; 49.4% men) who were free of AGA at baseline and had complete health screening data (Supplementary Fig 1, available via Mendeley at https://data.mendeley.com/datasets/t69fyny5mp/1). Long-term air pollution exposure was assessed as a time-varying variable by estimating average annual concentrations of PM_2_._5_ and PM_10_ using a high-resolution (1-km) Community Multiscale Air Quality model assigned to residential addresses (Supplementary Table I and Fig 2, available via Mendeley at https://data.mendeley.com/datasets/t69fyny5mp/1). Incident AGA was defined as ≥3 outpatient claims with an ICD-10 code (L64).

During a median 12.5-year follow-up, 5179 individuals developed AGA (Supplementary Table II, available via Mendeley at https://data.mendeley.com/datasets/t69fyny5mp/1). In fully adjusted time-varying Cox models, each 10-μg/m^3^ increase in PM_2_._5_ and PM_10_ was associated with a 44% (HR, 1.44; 95% CI, 1.30-1.59) and 61% (HR, 1.61; 95% CI, 1.52-1.71) higher risk of incident AGA, respectively (Table I). A clear dose-response relationship was observed; compared to the lowest concentration categories, AGA risk increased monotonically with higher PM levels (Fig 1). Notably, hazard ratios rose significantly even at “moderate” air-quality levels (PM_2_._5_: 20-25 μg/m^3^; PM_10_: 41-46 μg/m^3^), suggesting follicular susceptibility at traditionally acceptable exposure levels.^2^ In subgroup analyses, the risk associated with PM_2_._5_ was significantly higher in men than in women (HR, 1.61 vs 1.11; *P*_interaction_ = .006, Supplementary Table III, available via Mendeley at https://data.mendeley.com/datasets/t69fyny5mp/1). The robustness of these associations was confirmed through sensitivity analyses adjusting for psychiatric comorbidities and E-value estimation (Supplementary Table IV and Text, available via Mendeley at https://data.mendeley.com/datasets/t69fyny5mp/1).

**Table I:** Associations between PM long-term exposure (per 10 μg/m^3^) and risk of incident androgenetic alopecia

	PM_2.5_	PM_10_
Crude HR (95% CI)	1.65 (1.52-1.77)	1.94 (1.85-2.03)
Model 1 HR∗ (95% CI)	1.54 (1.42-1.66)	1.81 (1.72-1.89)
Model 2 HR† (95% CI)	1.43 (1.28-1.57)	1.60 (1.50-1.70)
Model 3 HR‡ (95% CI)	1.44 (1.30-1.59)	1.61 (1.52-1.71)

*95% CI*, 95% confidence interval; *HR*, hazard ratio.

∗ Model 1: Adjusted for age, sex.

† Model 2: Further adjusted for area, temperature, and relative humidity.

‡ Model 3: Further adjusted for income percentile, body mass index, smoking status, and metabolic syndrome.

**Fig 1:**
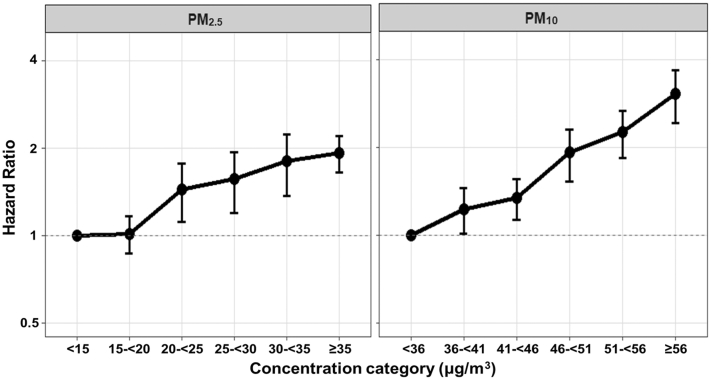
Dose-response plot for PM_2.5_ and PM_10_. *Dots* represent multivariable adjusted hazard ratios with 95% confidence intervals. For PM_2.5_, adjusted hazard ratios (95% CI) were 1.00 (reference) for concentrations <15 μg/m^3^, 1.02 (0.87-1.17) for 15 to <20 μg/m^3^, 1.44 (1.11-1.77) for 20 to <25 μg/m^3^, 1.57 (1.20-1.94) for 25 to <30 μg/m^3^, 1.80 (1.37-2.23) for 30 to <35 μg/m^3^, and 1.93 (1.65-2.21) for ≥35 μg/m^3^. For PM_10_, adjusted hazard ratios (95% CI) were 1.00 (reference) for concentrations <36 μg/m^3^, 1.23 (1.02-1.45) for 36 to <41 μg/m^3^, 1.34 (1.13-1.56) for 41 to <46 μg/m^3^, 1.92 (1.53-2.31) for 46 to <51 μg/m^3^, 2.26 (1.84-2.68) for 51 to <56 μg/m^3^, and 3.05 (2.42-3.68) for ≥56 μg/m^3^.

Biologically, PM-induced ROS can cause oxidative damage in follicular keratinocytes and dermal papilla cells, leading to premature catagen transition.^3^ PM components may also act as endocrine-disrupting chemicals, although direct effects on follicular androgen signaling remain unclear.^4^ Notably, AhR may interact with the androgen receptor pathway, providing a potential link between PM exposure and androgen-dependent miniaturization.^5^ Nevertheless, further mechanistic studies are warranted to elucidate whether PM directly exacerbates androgen-dependent miniaturization or induces a non-specific inflammatory phenotype that clinically mimics AGA.

The main limitations include potential underestimation of AGA occurrence due to claims-based outcome ascertainment, possible influence of healthcare-seeking behavior and exposure misclassification from residential-level exposure assignment, which cannot capture individual-level exposure variability. Nevertheless, the nationwide cohort design, high-resolution exposure modeling, and consistent dose–response patterns support the validity of the observed association. Furthermore, extensive sensitivity analyses further reinforce the consistency of these findings.

In conclusion, our findings provide population-level evidence that long-term ambient particulate matter exposure is associated with incident AGA. These findings support consideration of environmental exposure in alopecia research, and underscore the need for future studies to evaluate whether air-quality management provides clinical value in mitigating follicular damage.

## Conflicts of interest

None disclosed.
